# Time-of-flight resolved stimulated Raman scattering microscopy using counter-propagating ultraslow Bessel light bullets generation

**DOI:** 10.1038/s41377-024-01498-y

**Published:** 2024-07-01

**Authors:** Shulang Lin, Li Gong, Zhiwei Huang

**Affiliations:** Optical Bioimaging Laboratory, Department of Biomedical Engineering, College of Design and Engineering, National University of Singapore, Singapore, 117576 Singapore

**Keywords:** Multiphoton microscopy, Biophotonics, Imaging and sensing

## Abstract

We present a novel time-of-flight resolved Bessel light bullet-enabled stimulated Raman scattering (B^2^-SRS) microscopy for deeper tissue 3D chemical imaging with high resolution without a need for mechanical z-scanning. To accomplish the tasks, we conceive a unique method to enable optical sectioning by generating the counter-propagating pump and Stokes Bessel light bullets in the sample, in which the group velocities of the Bessel light bullets are made ultraslow (e.g., v_g_ ≈ 0.1c) and tunable by introducing programmable angular dispersions with a spatial light modulator. We theoretically analyze the working principle of the collinear multicolor Bessel light bullet generations and velocity controls with the relative time-of-flight resolved detection for SRS 3D deep tissue imaging. We have also built the B^2^-SRS imaging system and present the first demonstration of B^2^-SRS microscopy with Bessel light bullets for 3D chemical imaging in a variety of samples (e.g., polymer bead phantoms, biological samples such as spring onion tissue and porcine brain) with high resolution. The B^2^-SRS technique provides a > 2-fold improvement in imaging depth in porcine brain tissue compared to conventional SRS microscopy. The method of optical sectioning in tissue using counter-propagating ultraslow Bessel light bullets developed in B^2^-SRS is generic and easy to perform and can be readily extended to other nonlinear optical imaging modalities to advance 3D microscopic imaging in biological and biomedical systems and beyond.

## Abstract (web summary)

We report a novel time-of-flight resolved stimulated Raman scattering microscopy through counter-propagating ultraslow Bessel light bullets generation to realize deeper tissue 3D chemical imaging with high resolution.

## Graphical abstract

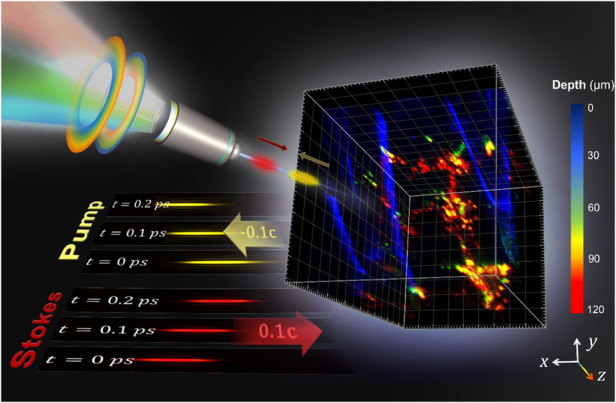

## Introduction

Stimulated Raman scattering (SRS) microscopy is an optical vibrational spectroscopic imaging technique that has emerged as an attractive label-free imaging tool for tissue and cell imaging and characterization with high biochemical specificity^1–4^. In conventional SRS (C-SRS) microscopy, the sample is illuminated by two tightly focused Gaussian beams (e.g., pump and Stokes beams) that are raster scanned across the sample to produce SRS 2D images. The SRS signal is obtained when the frequency difference of the pump and Stokes beams matches the specific vibrational frequency of the chemicals in the sample. However, the tightly focused Gaussian excitation beams used in C-SRS microscopy experience a strong light scattering effect that deteriorates the beam profile during propagation into turbid media (e.g., tissue) due to an inhomogeneous refractive index, leading to a degraded spatial resolution and limited light penetration incapable of volumetric deep tissue imaging^5,6^. To circumvent these problems, the use of non-diffracting beams (e.g., Bessel beam, Airy beam) has appeared as a promising alternative to improve penetration depth for 3D imaging^5^. However, raster scanning with Bessel beams can only provide SRS 2D projection images of the sample, and thus, the depth information is lost^7^. Approaches to retrieving 3D information in Bessel beam-based SRS imaging have been explored recently. For instance, SRS 3D information could be reconstructed by using Bessel beam-based stimulated Raman projection (SRP) tomography^8^. SRP relies on the traditional 360-degree mechanical rotation of a sample stage, which might introduce inertia artifacts that are not viable for in vivo rapid 3D imaging. Through generating optical beating patterns in the main lobe of Bessel beams, z-scanning-free stimulated Raman scattering tomography (SRST) can be realized in the frequency domain by using the optical beating technique (OBT)^9^, while the OBT-based SRST requires postprocessing, which might suffer from sample motion artifacts.

Time-of-flight techniques, such as light detection and ranging (LiDAR), are methods for measuring depth/distance information for macroscopic objects through direct measurement of the time-of-flight of light in space^10,11^. However, the conventional time-of-flight measurement is not possible for microscopic imaging due to its limited spatial resolution $\Delta z \sim {v}_{g}\Delta \tau$ in the millimeter scale, where ${v}_{g}$ is the group velocity of light (for collimated light beam in air, ${v}_{g}\approx c$, where $c$ (3 × 10^8^ m/s) is the speed of light in vacuum), and $\Delta \tau$ is the temporal resolution of the detector (e.g., single photon avalanche diode or streak camera) at least in the order of 10 picoseconds (ps)^12^. To push the LiDAR resolution $\Delta z$ up to the micrometer scale for microscopic imaging, we could consider some strategies: (i) for a light pulse made up of multiple plane wave components in both spatial and spectral domains, ${v}_{g}$ can be much slower than *c* by manipulating the angular dispersion^13–16^. Recently, a Bessel beam pulse with arbitrary group velocity in free space has been proposed theoretically through either a deformed pulse front^17^ or nanophotonic layer^18^, and generated experimentally using a combination of a spatial light modulator (SLM) and several phase plates^19^. (ii) If we measure the interaction between the two light pulses with different ${v}_{g}$ via a nonlinear optical process, the depth information whereby the nonlinear interaction^20,21^ takes place can be controlled by their relative speed or relative time-of-flight. In this case, the temporal resolution $\triangle \tau$ becomes the laser pulsewidth, e.g., hundreds of femtoseconds (fs) in typical SRS imaging. If the group velocity is ${v}_{g} \sim 0.1c$ and $\triangle \tau \sim 100\,{fs}$, $\triangle z$ is in the order of a few microns is possible for potential microscopy imaging. Therefore, the resolution of the time-of-flight-based techniques should be improved by at least three orders of magnitude in length for the mm-scale resolution of traditional LiDAR. However, the existing method^19^ of generating Bessel beam pulses with ${v}_{g}$ is only ~0.7*c* with a pulsewidth of ~ps, which is far lower than the micron- or sub-micron resolution requirement in microscopy imaging.

In this work, we present a novel time-of-flight resolved Bessel light bullet-enabled stimulated Raman scattering (B^2^-SRS) microscopy for deeper tissue SRS 3D chemical imaging. In B^2^-SRS, we conceive the unique dispersion control schemes that simultaneously convert both the pump and Stokes beam pulses into ultraslow Bessel light bullets ($\left|{v}_{g}\right| \sim 0.1c$) that are independently tunable in ${v}_{g}$ using a single SLM. We make the ultraslow pump and Stokes Bessel light bullets counter-propagating along the axial direction (i.e., pump: ${v}_{g,p} < 0$, Stokes: ${v}_{g,S} > 0$); thus, the depth-resolved SRS signal can be immediately detected by controlling the depth whereby the two Bessel light bullets meet with each other inside the sample through manipulating their relative time-of-flight without a need for mechanical z-scanning. We have comprehensively analyzed the generations of collinear multicolor ultraslow Bessel light bullets and experimentally demonstrated the utility of B^2^-SRS technique for label-free volumetric deeper tissue chemical imaging in a variety of samples (e.g., polymer bead phantoms, spring onion tissue, and porcine brain tissue) with high spatial resolution.

## Results

### Working principle of Bessel light bullet-enabled stimulated Raman scattering (B^2^-SRS) microscopy

#### Generation of ultraslow Bessel light bullets

**Fig. 1:**
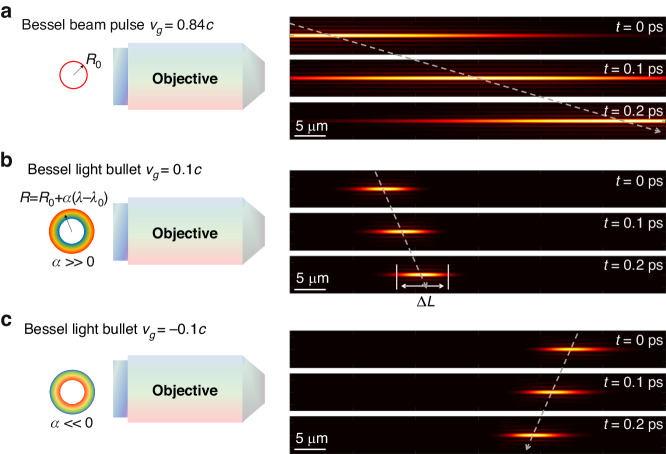
Principle of Bessel light bullet generation. **a** Propagation of a conventional Bessel beam pulse, of which the intensity distribution on the Fourier plane is a ring with the same radius for all wavelength components. **b**, **c** By introducing positive and negative angular dispersions on the Fourier plane, forward- and backward-propagating Bessel light bullets are generated. The generated Bessel light bullets have much slower group velocities and much shorter beam lengths than conventional Bessel beam pulses. The gray arrows indicate the propagation directions of the pulses

Figure 1 illustrates the mechanism of ultraslow Bessel light bullet generation using dispersion control. Figure 1a shows a conventional Bessel beam pulse, i.e., a Bessel beam pulse without dispersion. In the Fourier plane, the intensity distribution of all the wavelength components is a ring of the same radius ($R\left(\lambda \right)={R}_{0}$). Figure 1b, c shows Bessel beam pulses with a linear angular dispersion, of which $R$ is a linear function of the wavelength $\lambda$,

1 $$R\left(\lambda \right)={R}_{0}+\alpha (\lambda -{\lambda }_{0})$$

where ${\lambda }_{0}$ is the central wavelength and $\alpha$ is the angular dispersion coefficient.

The electric field distribution of a monochromatic Bessel beam of frequency $\omega$ is $E\left(r,z,t\right)={J}_{0}({krsin}\theta )\exp ({ikzcos}\theta -i\omega t)$, where $r$ and $z$ are the transverse and axial coordinates, respectively. ${J}_{0}$ is the zero-order Bessel function of the first kind, $\theta$ is the conical angle of the Bessel beam with respect to the $z$ axis, $k=n\frac{\omega }{c}$ is the wavevector of the light wave, and *n* is the refractive index^22^. For a Bessel beam pulse of spectrum $S(\omega )$, the electrical field distribution is the coherent summation of the electric field of each wavelength component:

2 $$\begin{array}{lll}E (r,z,t) &=& \displaystyle\int^{\infty}_{0} d \omega S (\omega) J_{0} [k (\omega) r {\rm{sin}} \theta] \, {\rm{exp}} [ik (\omega) z {\rm{cos}} \theta -i \omega t]\\ &=& \displaystyle\int^{\infty}_{0} d \omega S (\omega) J_{0} [k_{r} (\omega) r] \, {\rm{exp}} [ik_{z} (\omega) z - i \omega t ]\end{array}$$

The transverse and longitudinal components of the wavevector are:

3A $${k}_{r}(\omega )=k\left(\omega \right)\sin \theta$$

3B $${k}_{z}(\omega )=k\left(\omega \right)\cos \theta$$

In the presence of angular dispersion, $\theta$ is wavelength dependent,

4 $$\theta \left(\lambda \right)=\arcsin \frac{R\left(\lambda \right)}{f}$$

where $f$ is the focal length of the objective lens. Substituting Eqs. (1) and (4) into Eq. (3B) with $\omega =\frac{2\pi c}{\lambda }$, the group velocity of the Bessel beam pulse along the z-axis is

5 $${v}_{g}=\frac{d\omega }{d{k}_{z}}=\frac{c/n}{\cos {\theta }_{0}+\frac{\alpha {\lambda }_{0}}{f}\tan {\theta }_{0}}$$

where ${\theta }_{0}=\theta ({\lambda }_{0})$. Equation (5) shows that the group velocity is tunable by controlling the angular dispersion coefficient $\alpha$. A forward slowly propagating Bessel beam ($0 < {v}_{g}\ll \frac{c}{n}$) can be created if $\alpha \gg {\alpha }_{1} > 0$, with ${\alpha }_{1}=\frac{f\left(1-\cos {\theta }_{0}\right)}{{\lambda }_{0}\tan {\theta }_{0}}$. A backward slowly propagating Bessel beam ($-\frac{c}{n}\ll {v}_{g} < 0$) can be created if $\alpha \ll {\alpha }_{2} < 0$, with ${\alpha }_{2}=-\frac{{fcos}{\theta }_{0}}{{\lambda }_{0}\tan {\theta }_{0}}$.

Three cases of dispersion control are compared in Fig. 1, in which the simulation parameters are ${\lambda }_{0}=800\,{nm}$, $f=8\,{mm}$, ${R}_{0}=3.6\,{mm}$ and $n=1.33$ in water, so that ${\alpha }_{1}=2.13\times {10}^{3}$ and ${\alpha }_{2}=-1.77\times {10}^{4}$. Case 1 (Fig. 1a) represents the propagation of a conventional Bessel beam pulse without dispersion ($\alpha =0$), ${v}_{g}=\frac{c}{\left(n\cos {\theta }_{0}\right)}=0.84c$, which has the same order of magnitude of $c$. The shape of the conventional Bessel beam pulse is similar to a needle beam. Case 2 (Fig. 1b) shows a forward slowly propagating Bessel beam pulse ($\alpha =1.31\times {10}^{5} > > {\alpha }_{1}$, ${v}_{g}=0.1c$). Case 3 (Fig. 1c) shows a backward propagating Bessel beam pulse ($\alpha =-1.66\times {10}^{5}\ll {\alpha }_{2}$, ${v}_{g}=-0.1c$). In both Case 2 and Case 3, the Bessel beam pulses appear to shrink in the axial direction, propagating like a light bullet; thus, such a Bessel beam pulse is called as Bessel light bullet.

In B^2^-SRS, the length of the Bessel light bullet $\triangle L$ (labeled in Fig. 1b) is crucial to the optical sectioning capability, i.e., a shorter $\triangle L$ leads to a better axial resolution. According to Eq. (2), the intensity profile of the pulse at an arbitrary time along the z-axis is

6 $$I\left(0,z,t\right)={\left|E\left(0,z,t\right)\right|}^{2}\approx {\left|{\int_{0}}^{\infty }d\omega S\left(\omega \right){e}^{i\frac{z}{{v}_{g}}\omega -i\omega t}\right|}^{2}\propto {e}^{-\frac{4{ln}2}{{T}^{2}}{(\frac{z}{{v}_{g}}-t)}^{2}}$$

where the assumption ${k}_{z}\left(\omega \right)\approx {k}_{z}\left({\omega }_{0}\right)+\frac{1}{{v}_{g}}\left(\omega -{\omega }_{0}\right)$ is used, i.e., the group velocity dispersion (GVD) is ignored, since in our experimental conditions, the influence of GVD is negligible (refer to Supplementary Information S-1 for the calculation of GVD). Here, a Gaussian spectrum is assumed as $S\left(\omega \right)={e}^{-{T}^{2}({\omega -{\omega }_{0})}^{2}/8\mathrm{ln}2}$, where ${\omega }_{0}=\frac{2\pi c}{{\lambda }_{0}}$ and $T$ is the pulsewidth. Then, $\triangle L$ can be calculated as the full width at half maximum (FWHM) of the intensity profile along the axial direction:

7 $$\triangle L=\left|{v}_{g}\right|T$$

As a result, in Fig. 1a, a zero dispersion leads to ${v}_{g}\approx c$, thus resulting in a conventional needle-like Bessel beam pulse ($\triangle L=50\,\mu m$; here, $T=200\,{fs}$), while in Fig. 1b, c, the large angular dispersion leads to an ultraslow pulse ($\left|{v}_{g}\right|=0.1c$), thus resulting in ultrashort Bessel light bullets ($\triangle L=6\,\mu m$). Therefore, the light intensity distribution and the relative time-of-flight of ultraslow Bessel light bullets can be manipulated by controlling the angular dispersion of the light bullets to realize optical sectioning in tissue imaging.

#### B^2^-SRS imaging system

In SRS imaging, the sample is illuminated by the synchronized pump (${\omega }_{p}$) and Stokes $({\omega }_{s})({\omega }_{s} < {\omega }_{p}$) ultrafast laser pulses. If the frequency difference $({\omega }_{p}-{\omega }_{s}$) matches one of the Raman active vibrational modes of molecules in the sample, the SRS signal is generated where the two pulses are spatially and temporally overlapped. In B^2^-SRS, the pump and Stokes laser pulses are modulated as counter-propagating Bessel light bullets; thus, the depth-encoded SRS signal can be immediately retrieved by decoding the spatial information whereby the two Bessel light bullets meet in the sample for SRS 3D imaging.

**Fig. 2:**
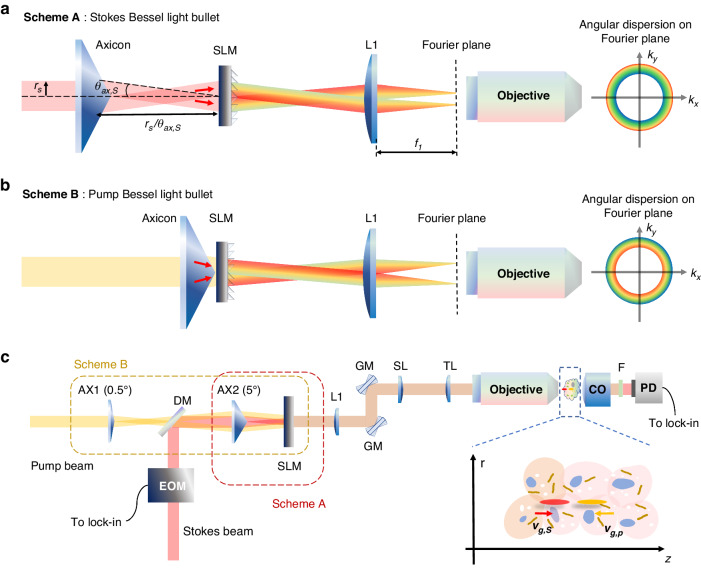
Schematic diagram of B^2^-SRS system for 3D imaging. **a**, **b** Schemes A and B to generate counter-propagating Stokes and pump Bessel light bullets with opposite angular dispersion. **c** Schematic of the B^2^-SRS imaging system, in which schemes A and B are implemented by using combinations of axicons and an SLM. AX axicon, EOM electro-optic modulator, DM dichroic mirror, SLM spatial light modulator, L lens, GM galvo mirror, CO condenser, PD photodiode, Lock-in Lock-in amplifier

Figure 2a, b illustrates dispersion control schemes A and B designed to generate counter-propagating Stokes and pump Bessel light bullets with positive and negative ${v}_{g}$, respectively. In scheme A, the Stokes beam is modulated sequentially by an axicon and an SLM, on which a blazed annular grating phase pattern with an inwards-blazed angle is displayed. In scheme B, the pump beam is modulated similarly, but the SLM displays an outwards-blazed annular grating phase pattern. Under the paraxial approximation, schemes A and B lead to opposite angular dispersions in the Fourier plane:

8A $${R}_{0S}={f}_{1}\left(\frac{{\lambda }_{0S}}{{d}_{S}}-{\theta }_{{ax},S}\right)$$

8B $${\alpha }_{S}=\frac{{f}_{1}}{{d}_{S}}$$

8C $${R}_{0p}={f}_{1}\left({\theta }_{{ax},p}-\frac{{\lambda }_{0p}}{{d}_{p}}\right)$$

8D $${\alpha }_{p}=-\frac{{f}_{1}}{{d}_{p}}$$

where ${f}_{1}$ is the focal length of lens L1; $d$ and ${\theta }_{{ax}}$ are the grating period and the deflection angle of the axicon, respectively (labeled in Fig. 2a); and the subscripts *“S”* and *“p”* stand for the Stokes and pump beams, respectively. To keep ${R}_{0S},{R}_{0p} > 0$, the grating periods should satisfy $\frac{{\lambda }_{0S}}{{d}_{S}} > {\theta }_{{ax},S}$ and $\frac{{\lambda }_{0p}}{{d}_{p}} < {\theta }_{{ax},p}$ for the Stokes and pump beams, respectively. The material dispersion of the axicon (fused silica) is ignored in Eq. (8), since it is orders of magnitude smaller than the grating dispersion, i.e., ${\theta }_{{ax},S}$ and ${\theta }_{{ax},p}$ are calculated by using the refractive index of the axicon at the central wavelength. Clearly, Eq. (8) shows that the dispersion is tunable by varying the grating period, while the radius of the ring in the Fourier plane is controlled by both the grating period and the axicon.

We further analyze the different dispersion control schemes developed. For instance, to achieve $\alpha \sim {10}^{5}$ with ${f}_{1} \sim {10}^{3}\,{mm}$, Eqs. (8B) and (8D) indicate that the grating period should be in the order of magnitude of ${d}_{S},{d}_{p} \sim 10\,\mu m$. However, if the axicon is removed, i.e., ${\theta }_{{ax},S}={\theta }_{{ax},p}=0$, Eqs. (8A) and (8C) indicate that the radius of the ring will be of the magnitude of ${R}_{0}={f}_{1}\frac{{\lambda }_{0}}{d} \sim {10}^{2}\,{mm}$ with ${\lambda }_{0} \sim 1\,\mu m$, which is much larger than the size of the optical components. Therefore, an axicon must be included to confine the radius of the ring inside the back aperture of the objective, with ${\theta }_{{ax},S},{\theta }_{{ax},p} \sim 0.1\,{rad}$.

In addition, the separation between the axicon and the SLM in scheme A is ${r}_{s}/{\theta }_{{ax},S}$, where ${r}_{s}$ is the Stokes beam radius before the axicon (labeled in Fig. 2a), while there is no separation in scheme B. This difference causes both pump and Stokes Bessel light bullets to be generated after the SLM, and thus, the pump and Stokes Bessel light bullets can encounter each other in the sample since they have overlapping propagation ranges for SRS generation.

Figure 2c depicts the experimental setup of the B^2^-SRS system built, which combines schemes A and B by using a single SLM. For the Stokes beam, scheme A is made up of an axicon (AX2: physical angle of 5°) and the SLM in a straightforward manner (red box with dashed boundary in Fig. 2c). For the pump beam, scheme B (brown box with dashed boundary in Fig. 2c) is implemented using the combination of the two axicons (AX1: physical angle of 0.5°, and AX2: physical angle of 5°), which is equivalent to a virtual axicon with a physical angle of 4.5° placed in contact with the SLM (refer to Supplementary S-2 for the equivalent virtual axicon). The phase pattern displayed on the SLM is made up of two annular gratings with different grating periods and opposite blazed angles for the Stokes and pump beams (refer to Supplementary S-3 for the phase pattern).

For SRS imaging, the Stokes beam is modulated at 20 MHz by using an electro-optic modulator (EOM) and combined with the pump beam via a dichroic mirror. The combined beams are delivered to the sample through a lens system (L1, scan lens, tube lens and objective lens) and raster scanned in the x-y plane by using galvo mirrors. Then, the pump beam is collected by a condenser after being filtered out by a set of bandpass filters and then detected by a large area photodiode. Finally, the SRS signal is demodulated by using a lock-in amplifier for B^2^-SRS imaging.

#### Mechanisms of optical sectioning in B^2^-SRS

**Fig. 3:**
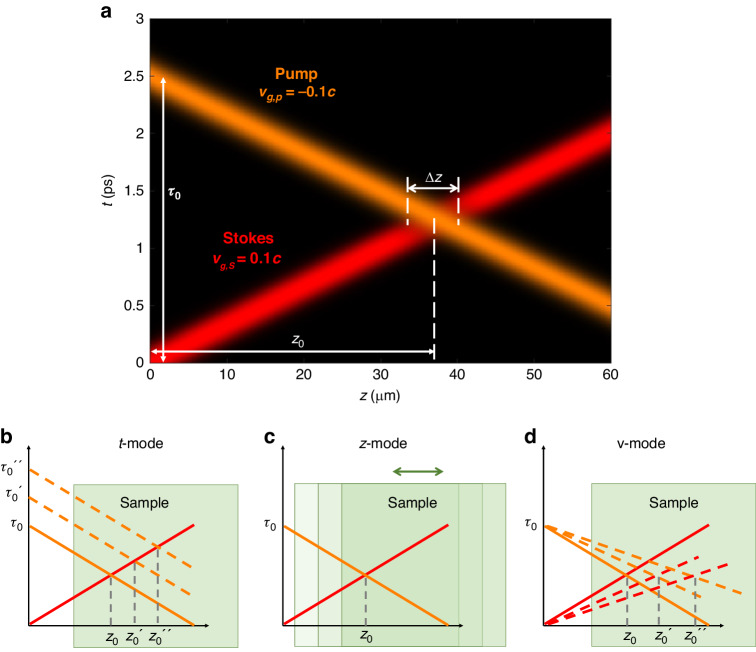
Mechanisms of optical sectioning in B^2^-SRS. **a** Counter-propagating ultraslow pump (brown color) and Stokes (red color) Bessel light bullets in the $t$-$z$ plane. The SRS is generated at the depth ${z}_{0}$ where the two counter-propagating Bessel light bullets meet. The depth resolution is determined by the overlapped region $\triangle z$. **b**–**d** represent the t-mode, $z$-mode, and v-mode, respectively, for B^2^-SRS 3D imaging

To illustrate the mechanisms of optical sectioning capability in B^2^-SRS, Fig. 3a shows the counter-propagations of the pump (in brown) and Stokes (in red) Bessel light bullets in the $t$-$z$ plane, as $I\left(0,z,t\right)$ in Eq. (6). With proper angular dispersion control (for details, refer to Supplementary S-3), the counter-propagating Bessel light bullets can travel at a speed much slower than the speed of light $c$. For a given relative time-of-flight ${\tau }_{0}$ between the pump and the Stokes laser pulses, the SRS signal is generated at the tissue depth where the two beams overlap between each other both spatially and temporally:

9 $$\begin{array}{lll}{I}_{{SRS}}\left(z,{\tau }_{0}\right)&=& \displaystyle\int {I}_{p}\left(0,z,t-{\tau }_{0}\right){I}_{S}\left(0,z,t\right){dt}=\int {e}^{-\frac{4\mathrm{ln}2}{{T}_{p}^{2}}{\left(\frac{z}{{v}_{g,p}}-t+{\tau }_{0}\right)}^{2}}{e}^{-\frac{4\mathrm{ln}2}{{T}_{S}^{2}}{\left(\frac{z}{{v}_{g,S}}-t\right)}^{2}}{dt}\propto \exp \left[-\frac{4\mathrm{ln}2}{{T}_{p}^{2}+{T}_{S}^{2}}{\left(\frac{z}{{v}_{r}}-{\tau }_{0}\right)}^{2}\right]\end{array}$$

where ${v}_{r}$ is the relative speed defined as $\frac{1}{{v}_{r}}=\left|\frac{1}{{v}_{g,S}}-\frac{1}{{v}_{g,p}}\right|=\frac{1}{\left|{v}_{g,p}\right|}+\frac{1}{\left|{v}_{g,S}\right|}$. The SRS signal is only generated in the overlapped region centered at ${z}_{0}={v}_{r}{\tau }_{0}$ (Fig. 3a), and thus the depth information can be uniquely encoded in time domain with the relative time-of-flight between the two Bessel light bullets by manipulating either ${v}_{r}$ or ${\tau }_{0}$, or the phase patterns. The axial resolution of B^2^-SRS can be calculated as the FWHM of the overlapped region, which can be obtained from Eq. (9):

10 $$\triangle {\rm{z}}={v}_{r}\sqrt{{{T}_{p}}^{2}+{{T}_{S}}^{2}}$$

There are three B^2^-SRS imaging modes to retrieve the depth information of the sample. That is, (i) t-mode (Fig. 3b): the SRS signals at different depths can be acquired by changing the relative time-of-flight ${\tau }_{0}$ between the pump and the Stokes pulses via a delay line; (ii) $z$-mode (Fig. 3c): the SRS signals at different depths can be obtained by simply moving the sample stage mechanically along the z direction (the same as in C-SRS), and (iii) v-mode (Fig. 3d): the SRS signals at different depths can also be acquired by changing the relative speed ${v}_{r}$ via ${v}_{g,p}$ and ${v}_{g,S}$ through varying the phase patterns displayed on SLM without a need of mechanical z-scanning. Note that in v-mode, the depth resolution $\triangle {\rm{z}}$ would vary at different depths that requires proper calibrations, as $\triangle {\rm{z}}$ ${is}$ a function of ${v}_{r}$ (Eq. (10)) (refer to Supplementary S-6 for details).

#### B^2^-SRS imaging of polymer beads in agarose gel phantoms, the relative group velocity and spatial resolutions

**Fig. 4:**
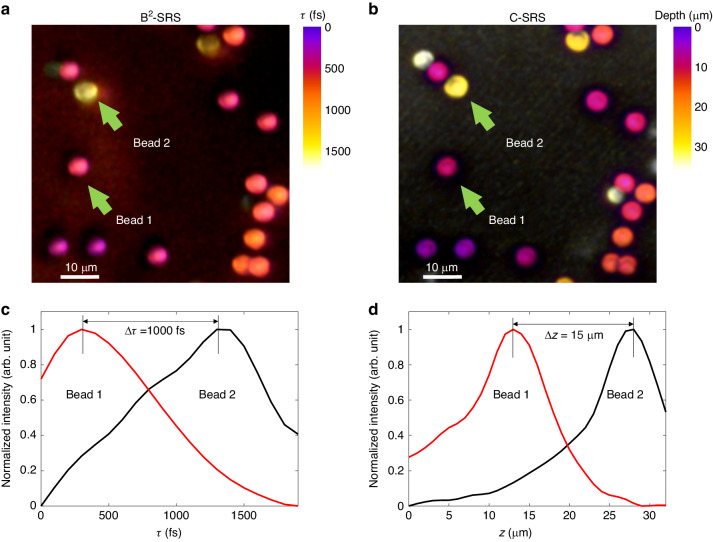
Measurements of the relative group velocity of v_*r*_. Comparison of t-mode B^2^-SRS (**a**) and C-SRS (**b**) images of 4.5 $\mu m$ PS beads in agarose gel phantoms. The intensity profiles of beads 1 and 2 along $\tau$-axis in B^2^-SRS (**c**) and along z-axis in C-SRS (**d**)

Figure 4a, b shows t-mode B^2^-SRS and C-SRS images on 4.5 $\mu m$ polystyrene (PS) beads embedded in agarose gel phantoms. The 3D distribution of PS beads obtained by t-mode B^2^-SRS is similar to the conventional point-scan C-SRS 3D imaging, confirming the z-scan-free optical sectioning of B^2^-SRS with Bessel light bullets. To measure the relative group velocity ${v}_{r}$ of the Bessel light bullets generated, Fig. 4c, d plot the intensity profiles of the two selected beads (e.g., bead 1 and bead 2 in Fig. 4a, b) along t-axis and z-axis in B^2^-SRS and C-SRS, respectively. The time delay t between the two beads is $\Delta \tau =1000\,{fs}$ (Fig. 4c), while the depth difference between the two beads is $\triangle z=15\,\mu m$ (Fig. 4d). Hence, we have ${v}_{r}=\frac{\triangle z}{\triangle \tau }=15\,\mu m{{ps}}^{-1}$. In our experiments, we set ${v}_{g,s}=-{v}_{g,p}={v}_{g}$, thus ${v}_{r}=\frac{{v}_{g}}{2}$ based on the relationship $\frac{1}{{v}_{r}}=\left|\frac{1}{{v}_{g,p}}-\frac{1}{{v}_{g,S}}\right|$. Hence, we obtain that ${v}_{g}=30\,\mu m{{ps}}^{-1}=0.1c$, confirming the group velocity v_g_ of the Bessel light bullets generated are substantially reduced for high resolution imaging.

**Fig. 5:**
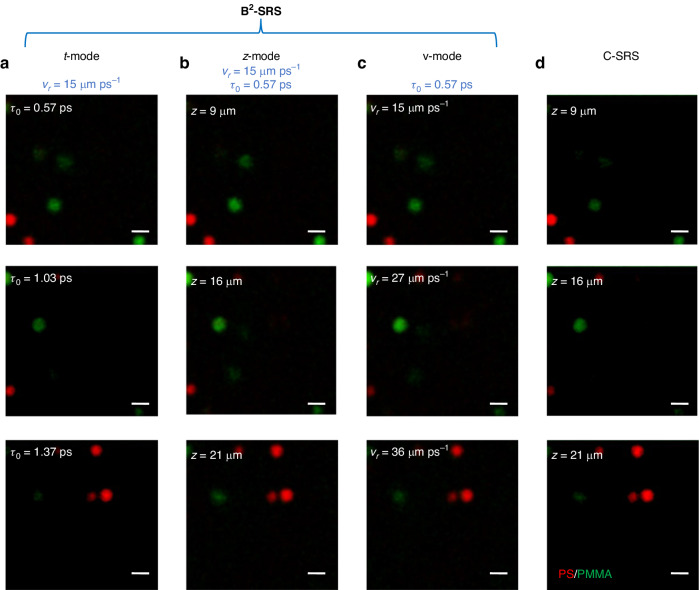
B^2^-SRS images of the mixed PMMA and PS beads embedded in agarose gel phantom. **a** t-mode, **b** z-mode, **c** v-mode of B^2^-SRS images, and **d** point-scan C-SRS images at different depths of selected volume of interest. PS (Raman shift of 3050 cm^−1^) in red; PMMA (Raman shift of 2950 cm^−1^) in green. The scalar bars are 10 $\mu m$

Figure 5a–c show the B^2^-SRS images of polymer beads embedded in agarose gel phantom ($4.5\,\mu m$ polystyrene (PS) beads (red), and $6\,\mu m$ polymethyl methacrylate (PMMA) beads (green)) acquired by using the t-mode, $z$-mode, and v-mode, respectively: (i) In t-mode (Fig. 5a), we keep ${v}_{r}=15\,\mu m{{ps}}^{-1}$ and keep the sample still while varying the relative time-of-flight ${\tau }_{0}$ as $0.57\,{ps}$, $1.03\,{ps}$ and $1.37\,{ps}$, corresponding to ${z}_{0}={v}_{r}{\tau }_{0}=8.6\,\mu m$, $15.5\,\mu m$ and $20.6\,\mu m$, respectively. (ii) In z-mode (Fig. 5b), we keep ${v}_{r}=15\,\mu {m}{{ps}}^{-1}$ and ${\tau }_{0}=0.57\,{ps}$, while the 3D SRS images are acquired by moving the sample stage mechanically along the z direction. (iii) In v-mode (Fig. 5c) with ${\tau }_{0}$ at $0.57\,{ps}$, through electronically varying the phase patterns on SLM while keeping the sample still, ${v}_{r}$ varies at 15 $\mu {m}{{ps}}^{-1}$, 27 $\mu {m}{{ps}}^{-1}$ and 36 $\mu {m}{{ps}}^{-1}$, corresponding to the predicted depths of ${z}_{0}={v}_{r}{\tau }_{0}=8.6\,\mu m$, $15.4\,\mu m$ and $20.5\,\mu m$, respectively. All the three imaging modes (i.e., t-mode, $z$-mode and v-mode) in B^2^-SRS give almost identical PS/PMMA bead distributions across different depths in the bead phantom compared to the C-SRS images of the same PS/PMMA bead phantom (Fig. 5d), confirming the optical sectioning ability of the B^2^-SRS technique in tissue imaging.

To further validate the z-scanning-free ability of v-mode-based B^2^-SRS for 3D imaging, we also monitored the 3D Brownian motions of 2 $\mu m$ PS beads in water with a volume rate of 10 vol s^−1^ (details refer to Supplementary Movie S1 in Supplementary S-7 in Supplementary Information). Supplementary Fig. S4 shows a snapshot of B^2^-SRS imaging (3050 cm^−1^) of 2 $\mu m$ PS beads in water obtained by electronically varying phase patterns on SLM with a maximum refresh rate of 60 Hz. In contrast, C-SRS imaging is unable to monitor Brownian motions due to the limited mechanical z-scanning speed of the sample stage or the objective lens (a few Hz).

**Fig. 6:**
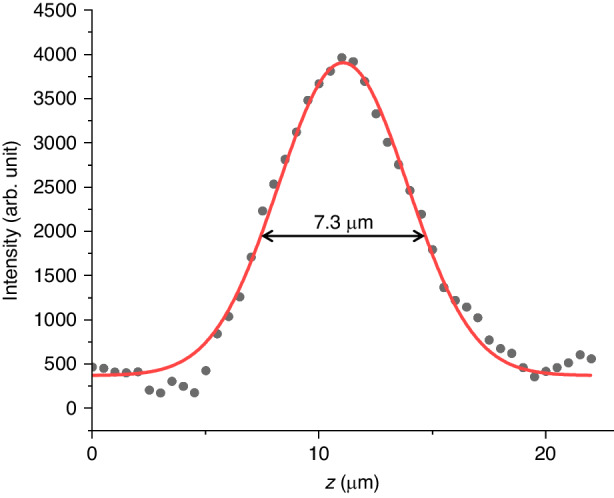
Axial resolution measurement of B^2^-SRS. The $5\,\mu m$ PMMA beads in an agarose gel phantom are measured with the z-mode B^2^-SRS (Raman shift of 2950 cm^−1^). The relative time-of-flight (${\tau }_{0}=0.57\,{ps}$) and the relative group velocity (${v}_{r}=15\,\mu {m}{{ps}}^{-1}$) are maintained

We also compared the axial and lateral resolutions between B^2^-SRS and C-SRS imaging. In B^2^-SRS, the axial resolution is determined by ${v}_{r}$ and pulsewidth according to Eq. (10). Figure 6 plots the z-intensity profile of B^2^-SRS image of a $5\,\mu m$ polymethyl methacrylate (PMMA) bead in an agarose gel phantom along the z-axis for ${v}_{r}=15\,\mu {m}{{ps}}^{-1}$. The measured full-width-at-half-maximum (FWHM) of the intensity profile is $7.3\,\mu m$. Considering the size of the PMMA bead ($5\,\mu m$), the actual axial resolution of B^2^-SRS can be calculated as $\sqrt{{7.3}^{2}-{5}^{2}}=5.3\,\mu m$ under *NA* = 0.6. The pulsewidths of the pump and Stokes beams on the sample are ${T}_{p}=120\,{fs}$ and ${T}_{S}=400\,{fs}$, respectively. According to Eq. (10), the predicted axial resolution $\triangle z={v}_{r}\sqrt{{{T}_{p}}^{2}+{{T}_{S}}^{2}}=6.3\,\mu m$, which is close to the measured resolution. In comparison with C-SRS using the same numerical aperture (NA), NA = 0.6, the FWHM of the Airy disk along the axial direction (2$\lambda$/NA^2^) are 4.44 $\mu m$ and 5.78 $\mu m$ for ${\lambda }_{p}=800\,{nm}$ and ${\lambda }_{S}=1041\,{nm}$, respectively. The axial resolution of C-SRS is $\frac{1}{\sqrt{1/{(4.44)}^{2}+1/{(5.78)}^{2}}}=3.5\,\mu m$. On the other hand, the lateral size of the Bessel light bullets ($\approx 612\,{nm}$) can be 1.3-fold higher^23^ than that of C-SRS ($\approx 813\,{nm}$, ${\lambda }_{p}=800\,{nm}$, NA = 0.6) in the sample. In addition, the depth of field (DOF) in the v-mode and t-mode is determined by the length of the Bessel beam, i.e., the propagation range of the Bessel light bullets (in tens of microns) (details refer to Supplementary S-4 for the propagation range), while the DOF in z-mode is only limited by the working distance of the objective lens (~1 ${mm}$). Hence, B^2^-SRS provides a higher lateral resolution and a comparable axial resolution with controllable DOF compared to C-SRS, enabling label-free 3D chemical imaging with a subcellular resolution.

#### Deep tissue volumetric imaging of biological samples

**Fig. 7:**
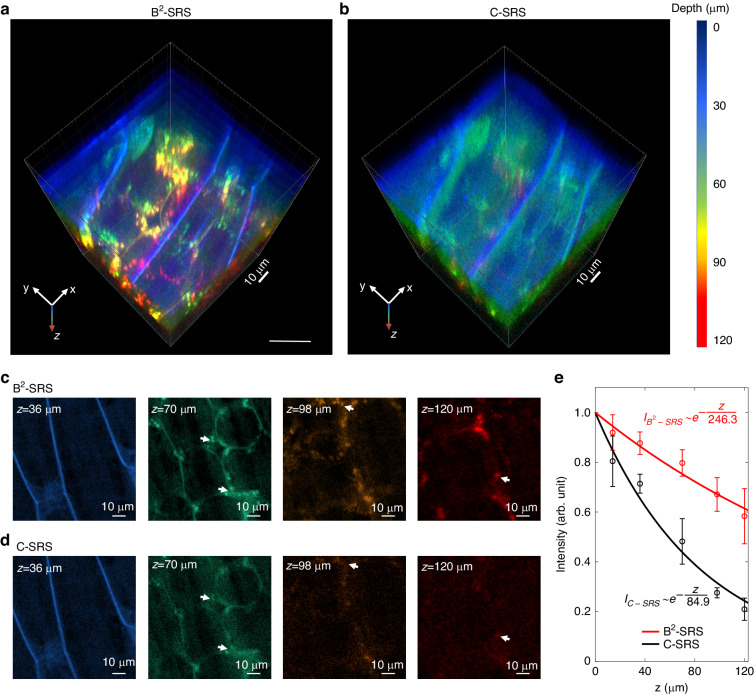
B^2^-SRS deep tissue imaging of spring onion tissue. Comparison of **a** B^2^-SRS (upper panel) and **b** C-SRS 3D images of spring onion tissue (Raman shift $2900\,{{cm}}^{-1}$). The depth information is represented by a pseudo-color scale. Comparison of **c** B^2^-SRS and **d** C-SRS images at different depths. **e** Normalized SRS intensities of B^2^-SRS and C-SRS in (**c)** and (**d)** as a function of tissue depth. The SRS intensity is normalized to the maximum intensity at the tissue subsurface. The error bar indicates the standard error

To demonstrate the advantage of the B^2^-SRS technique for deeper tissue 3D chemical imaging, Fig. 7a, b compares the z-mode B^2^-SRS image and the C-SRS image of spring onion tissue (C-H stretching of $2900\,{{cm}}^{-1}$ and two-photon absorption of chlorophylls in cell walls)^16^. Obviously, the cell walls and chloroplasts in deeper areas (in yellow and red colors) can be observed much more clearly with much brighter SRS signals and higher imaging contrast in B^2^-SRS. Figure 7c, d compares the B^2^-SRS images with C-SRS images at different tissue depths. In both B^2^-SRS and C-SRS images, the cell walls are clearly visualized at the shallower areas near the tissue subsurface (e.g., $z=\,36\,\mu m$). At deeper depths (e.g., $z=70\,\mu m,\,98\,\mu m$, chloroplasts (indicated by white arrows) are clearly observed in B^2^-SRS imaging, but this is not the case in C-SRS with a much dimmer signal and poorer image contrast, probably attributed to the strong scattering of the Gaussian beam used in tissue and thereby shorter imaging depth in C-SRS. At even deeper tissue depths (e.g., z > 120 $\mu m$), the SRS signal of chloroplasts can still be obtained in B^2^-SRS, whereas almost no signal could be detected in C-SRS. Figure 7e compares the SRS intensities from chloroplasts and cell walls vs imaging depth between B^2^-SRS and C-SRS imaging. The SRS intensity decay profiles plots are fit by an exponential decay function, $I={I}_{0}{e}^{-\frac{z}{\delta }}$, where δ is the penetration depth. For B^2^-SRS, $\delta =246.3\,\mu m$ (95% confidence interval [$142.3\,\mu m$, $350.3\,\mu m$]), while for C-SRS, $\delta =84.9\,\mu m$ (95% confidence interval [$49.0\,\mu m$, $120.8\,\mu m$]). The B^2^-SRS imaging technique provides an approximately 3-fold improvement in imaging depth compared to C-SRS imaging, substantiating that B^2^-SRS can be used for z-scanning-free deeper tissue 3D chemical imaging.

**Fig. 8:**
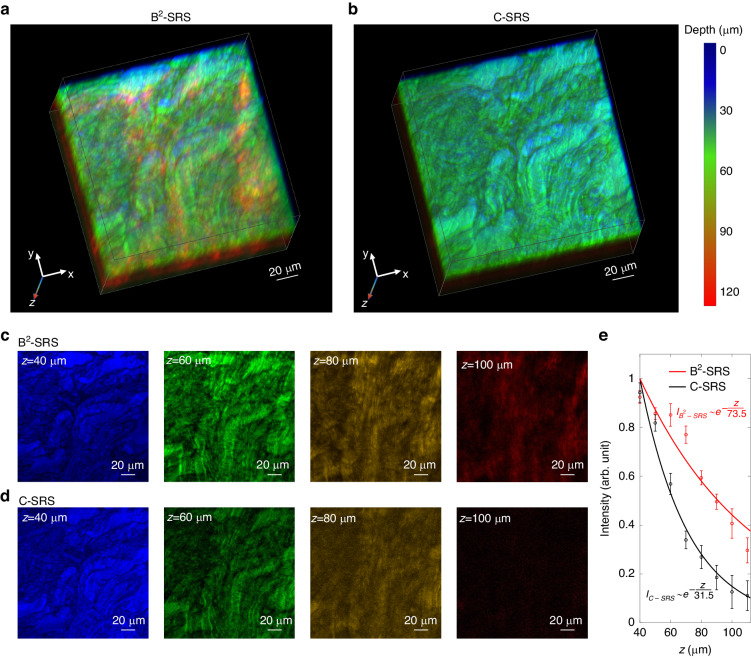
B^2^-SRS deep tissue imaging of porcine brain. Comparison of **a** B^2^-SRS and **b** C-SRS 3D images (Raman shift of $2935\,{{cm}}^{-1}$) of porcine brain tissue. The pseudo-color scale represents the depth information. **c**, **d** B^2^-SRS and C-SRS images of porcine brain tissue at different tissue depths. **e** Comparison of normalized intensity of SRS signals at different depths between B^2^-SRS and C-SRS in (**c**) and (**d**). Error bars represent the standard error

We have also applied B^2^-SRS to image fresh porcine brain tissue to further prove the ability of deeper tissue volumetric imaging. Figure 8a, b compares the B^2^-SRS and C-SRS images ($2935\,{{cm}}^{-1}$ of CH_3_ stretching of lipids and proteins) of the white matter in fresh porcine brain tissue. The complex morphology found by SRS imaging indicates that the white matter contains lipid- and protein-rich molecules, which are related to the functions in neuron communications between different parts of the brain^24^. Figure 8c, d compares the B^2^-SRS and C-SRS images at different brain depths. Both B^2^-SRS and C-SRS can generate comparable SRS signal levels at relatively shallower tissue regions (e.g., $z <\, 50\,\mu m$), while at deeper depths (e.g., z > 100 $\mu m$), the SRS signal can only be distinctly visible in the B^2^-SRS image but is barely detectable in the C-SRS image. To quantitatively analyze the penetration depth improvement in B^2^-SRS, Fig. 8e shows the normalized SRS intensities (with respect to the tissue subsurface z = 40 $\mu m$) at different depths in B^2^-SRS and C-SRS images. We again fit the SRS intensity profiles vs tissue depth by using the exponential decay function, and obtain that for B^2^-SRS, the penetration depth δ is $73.5\,\mu m$ (95% confidence interval [$48\,\mu m$, $99\,\mu m$]), while for C-SRS, $\delta =31.5\,\mu m$ (95% confidence interval [$25\,\mu m$, $38\,\mu m$]). Thus, B^2^-SRS gives a > 2-fold improvement in penetration depth in brain imaging compared to C-SRS imaging, further affirming the utility of B^2^-SRS for deeper tissue 3D chemical imaging.

## Discussion

Volumetric tissue imaging based on multimodal microscopy techniques (e.g., confocal microscopy, multiphoton microscopy, harmonic generation microscopy, and coherent Raman scattering microscopy)^25,26^ provides a global visualization of complex biosystems with more comprehensive information about 3D architectural structures of tissue and cells compared to 2D imaging, facilitating a better understanding of the fundamental mechanisms of live tissue and cell biology and biochemistry (e.g., in studies of cancer diagnosis and therapy, drug delivery, cell metabolism, neuron functions and developmental biology)^27–29^. In these conventional 3D imaging techniques (including C-SRS) based on tightly focused Gaussian beams, the strong light scattering effect in turbid media (e.g., tissue) dramatically attenuates light propagation in deeper tissue regions, limiting the imaging depth^30^. To overcome this issue, we developed an innovative B^2^-SRS imaging technique enabled by generating ultraslow Bessel light bullets for deeper tissue SRS 3D imaging. In B^2^-SRS, we have demonstrated an unprecedented strategy to convert the pump and Stokes beams into counter-propagating pump and Stokes Bessel light bullets with arbitrary group velocity, and optical sectioning is realized by implementing the relative time-of-flight control and detection scheme for deep tissue SRS 3D imaging. Hence, without the need for image postprocessing, the depth-resolved SRS signals can be directly acquired by controlling the spatiotemporal overlap of the ultraslow pump and Stokes Bessel light bullets generated in the sample, opening a new window for z-scanning-free SRS 3D imaging.

We have demonstrated the utility of B^2^-SRS for volumetric SRS deep imaging in a variety of samples (e.g., polymer beads, plant cells and porcine brain tissue) (Figs. 4–8). The Bessel light bullet generated in B^2^-SRS has shown a remarkable scattering resilience feature in turbid media, which is similar to the normal Bessel beam without angular dispersion^5^, enabling deeper SRS imaging in tissue. For instance, the deeper chloroplast constituents in onion tissue can still be clearly detected in B^2^-SRS (Fig. 7c) compared to C-SRS (Fig. 7d). Furthermore, for imaging porcine brain tissue with a much stronger scattering effect, the SRS signals of brain tissue in B^2^-SRS declined much slower at deeper depths than those in C-SRS imaging (Fig. 8e), proving the ability of B^2^-SRS for deeper tissue SRS 3D chemical imaging. Compared to other Bessel beam-based SRS imaging methods that apply optical beating^9^ or optical projection^8^ techniques to encode depth information spatially with acquisition of multiple raw images, B^2^-SRS provides z-sectioned image directly in time domain with relative time-of-flight controls without a need of images postprocessing for rapid deep-tissue 3D imaging.

In C-SRS and the t-mode and $z$-mode of B^2^-SRS for 3D imaging, the axial scanning speed is limited by the mechanical scans of the sample or the objective lens, or the delay line along the axial direction. Typically, mechanical actuation limits the axial scanning rate (mostly $\sim$10 Hz) with inertia artifacts. However, in v-mode B^2^-SRS imaging, the rapid z-scanning-free 3D imaging can be realized by electronically changing the phase patterns projected on the SLM (maximum rate of 60 Hz in the current experiments). We anticipate that the z-scanning speed in B^2^-SRS imaging could be further boosted if employing the state-of-the-art MEMS-based SLMs (a few kilohertzs) or a digital micromirror device (DMD) (tens of kilohertzs) with a refresh time of less than 1 ms^31^ for possible kHz SRS 3D imaging.

In comparison with other dispersion control methods (e.g., using a nanophotonic layer^18^ or using an SLM to introduce angular dispersion in 1D and several phase plates for the coordinate transformation to match circular symmetry^19^) to generate a forward-propagating distorted Bessel beam pulse, we proposed a unique angular dispersion control scheme using axicons and a SLM that is circularly symmetric modulated along the optical axis, thus allowing for multicolor collinear Bessel light bullets generation (e.g., Stokes and pump) without aberrations for high-resolution bioimaging. Our angular dispersion control design developed in B^2^-SRS imaging is robust and easy to operate, leading to a much reduced power loss of the excitation light beam with the cost of less GVD compensation for tissue and cell imaging. Nevertheless, the influence of GVD in B^2^-SRS is negligible. The GVD arising from the second-order term of Taylor’s expansion of ${k}_{z}\left(\omega \right)$ can be ignored as long as $\triangle R\ll {R}_{0}{\cos }^{2}{\theta }_{0}$, where $\triangle R=\alpha \triangle \lambda$, with bandwidth $\Delta \lambda =\frac{2{ln}2{\lambda }_{0}^{2}}{\pi {cT}}$. In our study, $\triangle R/\left({R}_{0}{\cos }^{2}{\theta }_{0}\right)\approx 0.25$; thus, such a condition is always satisfied (refer to Supplementary S-1 for the detailed calculation of GVD). For the case using a larger $\triangle R$, GVD needs to be compensated with proper spatiotemporal coupling to maintain a uniform axial resolution across different depths in tissue.

One notes that although the axial resolution reported in our current B^2^-SRS setting is ~$5.3\,\mu m$, the resolution can actually be further improved by optimizing the Bessel light bullet with a slower group velocity and shorter pulsewidth based on Eq. (10). In our experiments, the Stokes pulsewidth is chirped to ~400 fs. If the chirp can be compensated, the transform-limited (TL) Stokes pulse (170 fs) will improve the axial resolution to $3\,\mu m$ (NA = 0.6). Although Eqs. (9) and (10) in the main text is derived for TL pulses, Supplementary S-5 in Supplementary Information shows that in B^2^-SRS, the chirped pulse is equivalent to a TL pulse with the same pulsewidth. In addition, the group velocity as predicted by Eq. (6) can be arbitrarily slow, implying that the depth resolution can be arbitrarily high (Eq. (10)). However, in practice, the axial resolution of B^2^-SRS is eventually limited by the NA of the objective. In the case that the GVD is negligible, the depth resolution attained through a cross correlation of pump and Stokes Bessel light bullets cannot be higher than $\frac{1.4\lambda }{{{NA}}^{2}}$, which is close to the depth resolution of C-SRS imaging ($\frac{\sqrt{2}\lambda }{{{NA}}^{2}}$) (refer to Supplementary S-6 in Supplementary Information for the detailed analysis of the resolution limit). The axial resolution of B^2^-SRS could be further improved by twofold in a modified mode through incorporating a mirror-enhanced axial-narrowing super-resolution technique^32^, in which the pump beam is converted into two Bessel light bullets with different relative time-of-flight. Then one Bessel light bullet interferes with the other after reflecting by a mirror, creating a virtual 4Pi configuration with doubling the spatial frequency along the axial direction.

In summary, we have developed a unique time-of-flight resolved B^2^-SRS imaging technique for deeper tissue SRS 3D imaging without a need for mechanical z-scanning. We have comprehensively derived and analyzed the generation of counter-propagating ultraslow Bessel light bullets with the angular dispersion control schemes conceived, which open a new avenue to enable optical sectioning in SRS 3D imaging. We also built the B^2^-SRS imaging system and demonstrated the utility of the B^2^-SRS technique for label-free deeper 3D chemical imaging in a variety of samples (e.g., plant tissue cells and biological tissues). One notes that the optical sectioning method with ultraslow counter-propagating Bessel light bullet generation together with a flight-of-time resolved detection invented in B^2^-SRS is universal and can be readily applied to many other nonlinear optical imaging modalities, such as coherent anti-Stokes Raman scattering (CARS) microscopy^33^, sum-frequency generation (SFG) microscopy^34^ and higher-order CARS (HO-CARS)^35^. For nonlinear imaging modalities using only one laser beam (e.g., two-photon fluorescence (TPF) and second harmonic generation (SHG) microscopy), one can split the laser beam into two ultraslow counter-propagating Bessel light bullets. Similarly, the depth-resolved TPF/SHG signal can be directly generated from the detection of the flight-of-time resolved overlap between the two Bessel light bullets, but the background signal (DC component) caused by each Bessel light bullet should be subtracted. The concept of B^2^-SRS, which converts the temporal manipulation of Bessel light bullets to axial scanning in SRS 3D imaging, also provides new insights into the characterization of various dynamic space-time wave packets in 4D (e.g., including both spatial and temporal) with unprecedented spatiotemporal resolution and spectral information^36^. Further, the concept of time-of-flight resolved z-scanning modulating radial angular dispersion ($${k}_{r})$$ in B^2^-SRS can be extended for bi-directional time-dependent light steering^13^ of light bullets by applying both positive and negative angular dispersion controls in one lateral direction ($${k}_{x}$$ or $${k}_{y}$$). The relative time-of-flight control and detection scheme in B^2^-SRS enables continuous transverse scanning since the resolvable steering angle is limited by the temporal resolution of ultrafast detectors. Therefore, we anticipate that the unique time-of-flight resolved B^2^-SRS with counter-propagating ultraslow Bessel light bullets generation and tunable group velocity control will have broad applications for label-free deep tissue 3D chemical imaging in biological and biomedical systems and beyond.

## Materials and methods

### B^2^-SRS imaging system (Fig. 2c)

A dual output ultrafast laser (Insight DeepSee+, Spectra-Physics) operating at a repetition rate of 80 MHz is used as the excitation source in SRS imaging. The fixed output at 1040 nm and a tunable output (680~1300 nm) serve as the Stokes and pump beams, respectively, for B^2^-SRS imaging. The transform-limited (TL) pulsewidths of the pump and Stokes beams are 120 fs and 170 fs, respectively. In our experiments, the pump beam pulsewidth remains 120 fs in the sample since there is a pre-chirping setting for the pump beam inside the laser cavity to compensate for the chirp induced in the optical path of the B^2^-SRS system, while the Stokes pulsewidth is chirped to ~400 fs in the sample due to multiple optical components in the optical path (refer to Supplementary S-5 for details on how the chirping affects the axial resolution of B^2^-SRS imaging). The Stokes beam is modulated at 20 MHz by an electro-optic modulator (EOM) (APE, GmbH, Berlin). The pump beam is first modulated by an axicon (physical angle of $0.5^{\circ}$, AX2505-B, Thorlabs) and is combined with the Stokes beam by a dichroic mirror. Then, both the pump and Stokes beams pass through another axicon (physical angle of $5^{\circ}$, AX255-B) and impinge onto the SLM (Meadowlark E-series 1920 × 1200). The annular rainbow patterns on the focal plane of L1 (${f}_{1}=500\,{mm}$) are projected onto the back aperture of a water immersion microscope objective lens (Apo LWD 25X, NA = 1.10, Nikon) through a 4-f system composed of a scan lens and a tube lens in the microscope (MPM-4R, Thorlabs). An oil condenser (CC Achromat/Aplanat, NA = 1.4, Nikon) is used to collect the transmitted pump beam, which is isolated from the Stokes beam with a set of bandpass filters (Semrock). A large area photodiode (APE, Berlin) coupled with a lock-in amplifier (APE, Berlin) is used to detect the stimulated Raman loss (SRL) signal from the sample for SRS 3D imaging.

### Imaging parameters

(i) Laser power: The average power on the samples is 35 mW for the pump beam and 40 mW for the Stokes beam in B^2^-SRS and 10 mW for the pump beam and 20 mW for the Stokes beam in C-SRS. These average powers were chosen based on the consideration of achieving the same signal-to-noise ratio (SNR) in the surface of the samples for both B^2^-SRS and C-SRS. (ii) Imaging time: For PS/PMMA beads imaging, the pixel dwell time used for both B^2^-SRS and C-SRS is 8 μs. For spring onion tissue, the pixel dwell time is 16.8 μs. For porcine brain tissue, the pixel dwell time is 14 μs. Such pixel dwell times are selected to ensure SRS imaging to achieve SNR levels of >10 in the samples for high-quality SRS 3D imaging. The pixel number is 256 × 256 with a pixel size of 360 nm. In the z-mode B^2^-SRS and C-SRS, the pixel size along the axial direction is 2 μm. In the t-mode B^2^-SRS, the step size of the delay scanning with an actuator is 10 μm, corresponding to a step size of the relative time-of-flight of 66.7 fs.

### Sample preparations

The mixed PMMA and PS beads in the agarose phantom: The 6$\,\mu m$ PMMA beads and 4.5 $\mu m$ PS beads are embedded in a 2 wt% agarose gel phantom for B^2^-SRS and C-SRS imaging. The refractive index of the agarose gel phantom is ~1.33. The SRS signal of PMMA beads is detected at 2950 cm^−1^, while PS beads generate SRS signals at both 2950 cm^−1^ and 3050 cm^−1^. The SRS intensity ratio of PS beads at 2950 cm^−1^ and 3050 cm^−1^ is ~0.8:1. The SRS image of PMMA beads is obtained by subtracting the SRS image at 2950 cm^−1^ (${I}_{2950}$) of PMMA/PS beads from the SRS image at 3050 cm^−1^ (${I}_{3050}$) of PS beads multiplied by a factor of 0.8 (i.e., ${I}_{2950}-{0.8\times I}_{3050}$). The SRS signal of agarose is much weaker than those of PMMA and PS beads due to its low concentration ($\sim2$ wt%), which can be neglected. Bio-samples: Spring onion and porcine brain tissue samples were acquired from a local supermarket.

### Supplementary information

Supplementary Information

Movie S1
