# Supplementary material for: Time-of-flight resolved stimulated Raman scattering microscopy using counter-propagating ultraslow Bessel light bullets generation
Source: Light Sci Appl. 2024 Jul 1;13:148. doi: 10.1038/s41377-024-01498-y (PMC11217417; doi:10.1038/s41377-024-01498-y)
Supplement: Supplementary file 1 — Supplementary Information [file 41377_2024_1498_MOESM1_ESM.docx]

**Supplementary Information for “Time-of-flight resolved stimulated Raman scattering microscopy using counter-propagating ultraslow Bessel light bullets generation”**

Shulang Lin^1^, Li Gong^1^, Zhiwei Huang^1*^

*^1^Optical Bioimaging Laboratory, Department of Biomedical Engineering, College of Design and Engineering, National University of Singapore, Singapore 117576*

* Corresponding author: [biehzw@nus.edu.sg](mailto:biehzw@nus.edu.sg)

**S-1. Analysis of influence of the group velocity dispersion (GVD) on propagation of the Bessel light bullets**

In the derivation of Eq. (6) in the main text, we assume $k_{z}\left( \omega\right)\approx k_{z}\left( \omega_{0} \right)+\frac{1}{v_{g}}\left( \omega-\omega_{0} \right)$ by neglecting the higher order terms, which stands for GVD. In S-1, firstly, we analyze the influence of the GVD in the propagation of Bessel light bullet in general. Then, a criterion is proposed to analyse whether GVD can be neglected. Finally, we prove that such a criterion is satisfied in our experimental conditions.

***S-1.1 Bessel light Bullet propagation with GVD***

To include the GVD term, we expand $k_{z}\left( \omega\right)$ as

$k_{z}\left( \omega\right)\approx k_{z}\left( \omega_{0} \right)+\frac{1}{v_{g}}\left( \omega-\omega_{0} \right)+\frac{k_{z}"}{2}\left( \omega-\omega_{0} \right)^{2}$ (S1-1)

where $k_{z}"=\frac{d^{2}}{{d\omega}^{2}}k_{z}=\frac{d}{d\omega}\frac{1}{v_{g}}$ reflects the GVD. Substitute Eq. (S1-1) into Eq. (2) with $S\left( \omega\right)=e^{-T^{2}({\omega-\omega_{0})}^{2}/8ln2}$, we can re-derive Eq. (6) as

$$I\left( 0,z,t \right)=\left| E\left( 0,z,t \right) \right|^{2}\approx\left| \int_{-\infty}^{\infty} e^{-\left( \frac{T^{2}}{8ln2}-i\frac{k_{z}"}{2}z \right){\omega'}^{2}}e^{i\left( \frac{z}{v_{g}}-t \right)\omega'}d\omega' \right|^{2}$$

$\propto e^{-\frac{4ln2}{T^{2}}{(\frac{z}{v_{g}}-t)}^{2}\left[ \frac{1}{1+\left( \frac{4ln2}{T^{2}}k_{z}"z \right)^{2}} \right]}$ (S1-2)

where $\omega'=\omega-\omega_{0}$. It shows that the length of the Bessel light bullet will be broadened as it propagates away from the origin,

$\Delta L=\left| v_{g} \right|T\sqrt{1+\left( \frac{4ln2}{T^{2}}k_{z}"z \right)^{2}}$ (S1-3)

Thus, GVD can be neglected, i.e., the Bessel light bullet is not obviously broadened, only when $\left| z \right|$ is small enough,

$z<z_{max}=\frac{T^{2}}{4ln2\left| k_{z}" \right|}$ (S1-4)

where $z_{max}$ is the location where the Bessel light bullet is broadened $\sqrt{2}$-fold. Therefore, $z_{max}$, within which the GVD is negligible, can be consider as the GVD-free propagation range.

***S-1.2 Criterion of negligible GVD***

The optical sectioning ability of B^2^-SRS is determined by the length of the Bessel light bullet $\Delta L$, while the depth-of-view (DOV) is determined by $z_{max}$, thus, a proper SRS 3D image of the sample can be acquired only when $\Delta L\ll z_{max}$. From Eq. (S1-3) and Eq. (S1-4), such a criterion implies

$\frac{4ln2\left| v_{g}k_{z}" \right|}{T}\ll1$ (S1-5)

According to Eqs. (1), (3b) and (4) in the main text, we first compute $k_{z}"$ as

$k_{z}"=-\frac{n}{c}\frac{\lambda_{0}^{2}}{2\pi c}\frac{\lambda_{0}\alpha^{2}}{f^{2}\cos^{3} \theta_{0}}$ (S1-6)

According to Eq. (5) in the main text, under the condition of $v_{g}\ll c$ (in our experiments, $\left| v_{g} \right|\sim0.1c$), we have

$v_{g}=\frac{c/n}{cos\theta_{0}+\frac{\alpha\lambda_{0}}{f}tan\theta_{0}}\approx\frac{c/n}{\frac{\alpha\lambda_{0}}{f}tan\theta_{0}}$ (S1-7)

Substitute Eqs. (S1-6) and (S1-7) into (S1-5), the criterion becomes

$\frac{4ln2}{T}\frac{\lambda_{0}^{2}}{2\pi c}\frac{\alpha}{f\sin\theta_{0}\cos^{2} \theta_{0}}\ll1$ (S1-8)

Eq. (S1-8) can be interpreted in physical picture by using the relationship between the pulsewidth $T$ and the thickness $\Delta R$ of the annular rainbow in Figs. 1(b-c). From, Eq. (1) in the main text, we have $\Delta R=\alpha\Delta\lambda$, where $\Delta\lambda=\frac{\lambda_{0}^{2}}{2\pi c}\Delta\omega$ is the bandwidth of the Bessel light bullet. Since $\Delta\omega=\frac{4ln2}{T}$ , we have

$\alpha\frac{\lambda_{0}^{2}}{2\pi c}\frac{4ln2}{T}=\alpha\frac{\lambda_{0}^{2}}{2\pi c}\Delta\omega=\alpha\Delta\lambda=\Delta R$ (S1-9)

Substitute Eq. (S1-9) into Eq. (S1-8) and note that $f\sin\theta_{0}=R_{0}$, it shows that

$\Delta R\ll R_{0}\cos^{2} \theta_{0}$ (S1-10)

Similarly, $z_{max}$ in Eq. (S1-4) can be simplified as

$z_{max}=ln2\left( \frac{\Delta R}{R_{0}} \right)^{2}\frac{\lambda_{0}}{n}\frac{\cos\theta_{0}}{\tan^{2} \theta_{0}}$ (S1-11)

Hence, if the thickness of the rainbow $\Delta R$ is much less than its radius $R_{0}$ multiplied by $\cos^{2} \theta_{0}$, the GVD can be neglected in B^2^-SRS; the GVD-free propagation range can be calculated by Eq. (S1-11).

***S-1.3 GVD-free propagation range in experimental conditions***

In our experiments, $\Delta R\approx0.8 mm$, while $R\approx4 mm$ and $\cos^{2} \theta_{0}=0.80$, thus the condition $\Delta R\ll R_{0}\cos^{2} \theta_{0}$ is always satisfied. It implies that the GVD can be neglected in our experimental conditions. According to Eq. (S1-11), the GVD-free propagation range $z_{max}\approx40 \mu m$.

**S-2. Optical design of the dispersion scheme for the pump beam in B^2^-SRS**

An axicon refracts the incident light at the physical angle with respect to the optical axis. The phase function $\varphi\left( r \right)$of an axicon can be given by

$\varphi\left( r \right)=\left( n-1 \right)rsin\beta_{ax}$(S2-1)

where n is the refractive index of the material of the axicon, r is the transverse coordinate, and $\beta ax$ is the physical angle of the axicon. According to Eqs. S1-11 and Eq. S2-1, the phase function of an axicon is linear depending on the r. In Fig. S1(a), the pump beam passes through a 0.5-degree axicon first before entering the 5-degree axicon, and thus the incident angle on the 5-degree axicon is considered as negative. Hence, the phase imposed to the pump light after the 5-degree axicon is $\varphi_{0.5}+\varphi_{5}=\left( n-1 \right)r(-sin\beta_{ax,0.5}+sin\beta_{ax,5})$, where the subscription ‘0.5’ and ‘5’ represents the phase and reflection angles induced by the 0.5-degree and 5-degree axicons, respectively. We note $\beta_{ax0.5}=0.0087\approx sin\beta_{ax,0.5}$ and $\beta_{ax5}=0.087\approx sin\beta_{ax,5}$. Hence, the combination of a 0.5-degree and a 5-degree axicon in Fig. S1(a) is equivalent to an axicon of 4.5-degree (Fig. S1(b)).

According to the dispersion scheme B (Fig. 2(b) in the main text) for the pump Bessel light bullet, an axicon should be placed in contact with the spatial light modulator (SLM). However, in practice, the distance between the axicon and the SLM cannot be zero, thus we adopt an equivalent scheme as shown in Fig. S1, in which the combination setting of a 0.5-degree and a 5-degree axicons placed a slightly away from a SLM to represent a 4.5-degree in contact with the SLM.


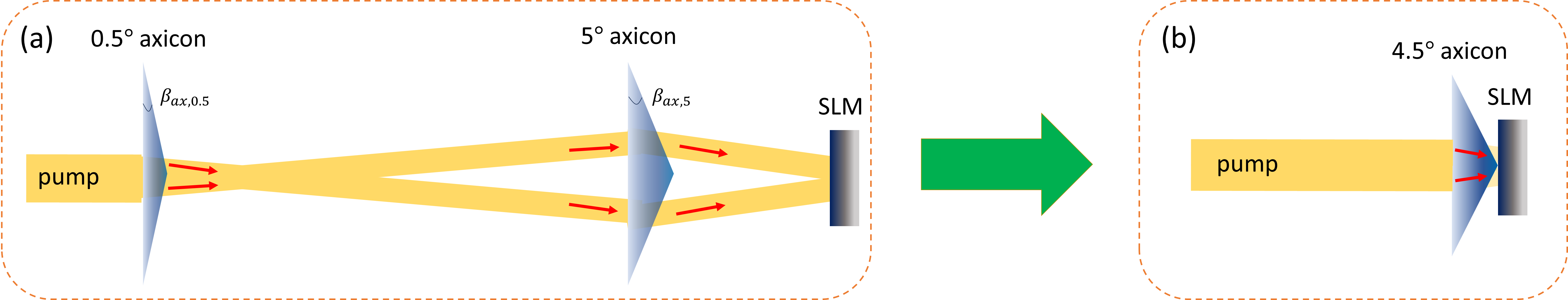


**Figure S1. Equivalent dispersion scheme for the pump beam. (a)** the combination of a 0.5-degree axicon and a 5-degree axicon placed slightly away from a SLM is equivalent to **(b)** a 4.5-degree axicon in contact with a SLM.

**S-3. The phase pattern on the SLM for pump and Stokes Bessel light bullets**

In B^2^-SRS, a spatial light modulator (SLM) (15.4 mm × 9.6 mm active area, 1920 × 1200 pixels) is used to modulate both pump and Stokes beams. As shown in Figure S2, the phase pattern displayed on the SLM is a combination of the phase patterns for both beams. Specifically, the SLM is divided into 128 sector regions, which are labelled by integers in sequence. The sector regions with odd and even number labels display the phase patterns for the pump and Stokes beams, respectively.

Both pump and Stokes beams require phase pattern of a blazed annular grating, in the form of $\varphi_{p,S}=K_{p,S}\times r$. In Fig. S2, $K_{p}=-0.2626 {\mu m}^{-1}$ and $K_{S}=0.2505 {\mu m}^{-1}$, of which the minus and plus signs indicating that the blazed angles for the pump and Stokes beams are outwards and inwards, respectively. Eventually, the pump and Stokes Bessel light bullets are in opposite group velocities, i.e., $v_{g,p}\approx-0.1c$and $v_{g,S}\approx0.1c$, respectively.


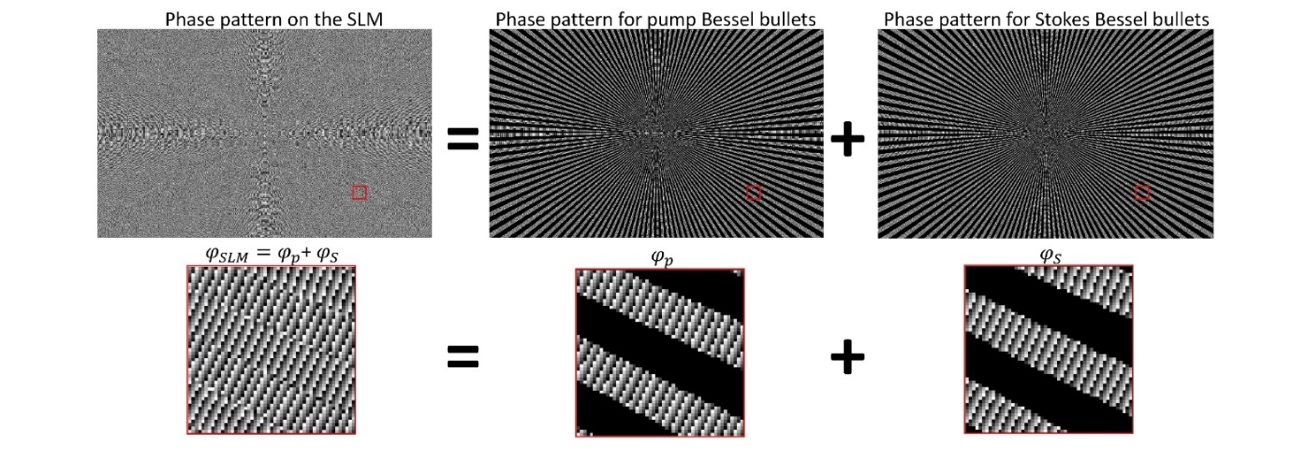


**Figure S2. Phase patterns displayed on the SLM.** (Upper panel) Phase pattern displayed on the SLM is a combination of the phase patterns for pump and Stokes beams. (Lower panel) Enlarged images of the selected areas (red box in the upper panel, pixel size of 50$\times$50).

**S-4. Propagation range of the Bessel light bullets**

The propagation range of the Bessel light bullets is determined by the length of the Bessel beam, which is controlled by the beam size before the axicon, the parameters of the axicons, the phase pattern displayed on the SLM lenses and the objective.

To measure the propagation ranges of both the pump and Stokes Bessel light bullets, Figure S3(a) shows the two-photon fluorescence (TPF) images of a 6 $\mu m$ fluorescent bead in x-z plane, under the excitation of pump Bessel light bullet ($v_{g,p}\approx-0.1c$) and Stokes Bessel light bullet ($v_{g,S}\approx0.1c$), respectively. Since the pixel dwell time (in the order of $\sim\mu s$) used in B^2^-SRS imaging is much longer than the propagation time of the Bessel light bullet from one end of the Bessel beam to the other end (in the order of $\sim ps$), the images we acquired are the time-lapse images of the Bessel light bullets. Figure S3(b) plots the square root of the intensity profiles ($\sqrt{I_{TPF}}$) along the dashed white lines in Fig. S3(a). $\sqrt{I_{TPF}}$ is chosen, because the TPF signal is proportional to the square of excitation intensity, i.e., $I_{TPF}\sim I_{exc}^{2}$. Hence, the propagation ranges can be measured based on the full width at half maximum (FWHM) of $\sqrt{I_{TPF}}$, which are $26 \mu m$ and $34 \mu m$, respectively, for the pump and Stokes Bessel light bullets.


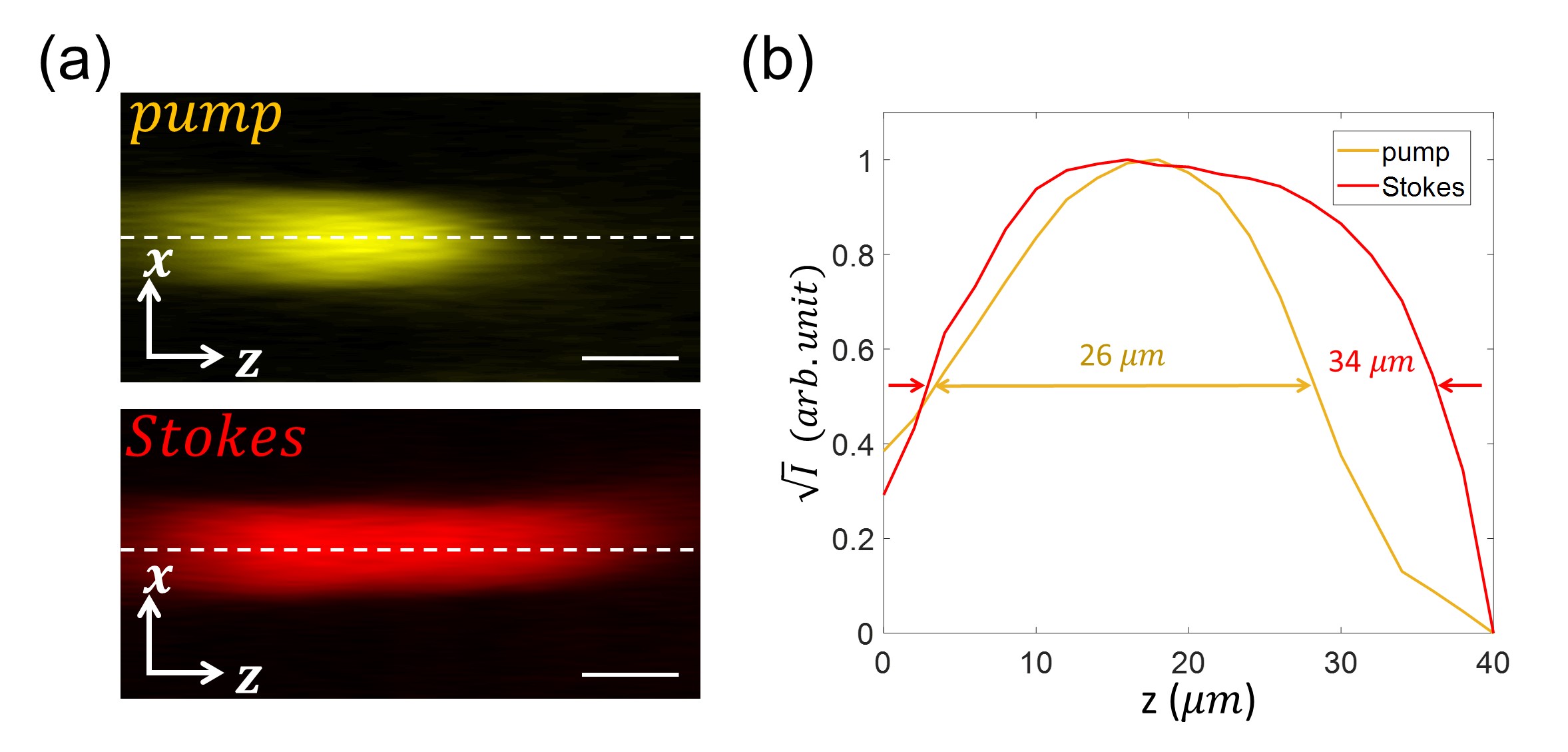


**Figure S3. Propagation range of Bessel light bullets.** (a) TPF images of a $6 \mu m$ fluorescent bead excited by pump (upper panel) and Stokes Bessel light bullets (lower panel), respectively. The scale bar represents 10$\mu m$. (b) The square root of the intensity profiles along the dashed white lines in (a). The propagation ranges of the pump and Stokes Bessel light bullets in the sample are given in (b), respectively.

**S-5. Bessel light bullet generation by the chirped pulse**

In our experiments, the transform limited (TL) Stokes pulsewidth is 170 fs. However, it is chirped to ~400 fs in the sample, due to multiple optical components in the beam path. The pump pulse is still TL in the sample (120 fs), since the chirp induced by the optical components is compensated by a pre-compensation system inside the laser source.

Here, we prove that the length of the Bessel light bullet generated by a chirped pulse is equal to the length of the Bessel light bullet generated by a TL pulse of the same pulsewidth as the chirped pulse.

For a chirped pulse, the spectrum is

$S\left( \omega\right)=e^{-\left( \frac{T^{2}}{8ln2}-i\gamma\right)({\omega-\omega_{0})}^{2}}$ (S5-1)

where $T$ is its TL pulsewidth and $\gamma$ reflects the chirp. Substitute Eq. (S5-1) into Eq. (6) in the main text (GVD is neglected), the propagation of chirped Bessel light bullet is

$$I\left( 0,z,t \right)=\left| \int_{0}^{\infty} S(\omega)e^{i\left( \frac{z}{v_{g}}-t \right)\omega}d\omega\right|^{2}\approx\left| \int_{-\infty}^{\infty} e^{-\left( \frac{T^{2}}{8ln2}-i\gamma\right){\omega'}^{2}}e^{i\left( \frac{z}{v_{g}}-t \right)\omega'}d\omega' \right|^{2}$$

$\propto e^{-\frac{4ln2}{T^{'2}}{(\frac{z}{v_{g}}-t)}^{2}}$ (S5-2)

where $T'=T\sqrt{1+\left( \frac{8ln2}{T^{2}}\gamma\right)^{2}}$ is the chirped pulsewidth. Compare Eq. (S5-2) with Eq. (6) in the main text, it shows that the intensity distribution of the chirped Bessel light bullet is identical to that of a TL Bessel light bullet of pulsewidth $T^{'}$. So does the length of the Bessel light bullet $\Delta L=\left| v_{g} \right|T'$.

**S-6. Analysis of axial resolution limit of B^2^-SRS imaging**

The axial resolution of B^2^-SRS is given in Eq. (10) in the main text, the axial resolution can be arbitrarily high in principle, as we can always choose a shorter pulsewidth and make the Bessel bullet slower. However, the condition of negligible GVD (Eq. (S1-10)) imposed a lower limit to the product of pulsewidth $T$ and group velocity $v_{g}$.

In our experiments, we set $v_{g,S}=-v_{g,p}=v_{g}>0$, thus the relationship $\frac{1}{v_{r}}=\left| \frac{1}{v_{g,pS}}-\frac{1}{v_{g,Sp}} \right|$ implies that

$v_{r}=v_{g}/2$ (S6-1)

If we combine Eqs. (S1-7) (S1-9) and (S6-1), and assume $T_{p}=T_{S}=T$, the axial resolution can be expressed:

$$\Delta z=\frac{v_{g}T}{\sqrt{2}}\approx\frac{1}{\sqrt{2}}\times\frac{\frac{c}{n}}{\frac{\alpha\lambda_{0}}{f}\tan\theta_{0}}\times\alpha\frac{\lambda_{0}^{2}}{2\pi c}\frac{4ln2}{\Delta R}$$

$=\frac{\sqrt{2}ln2}{n\pi}\frac{f\lambda_{0}}{\Delta R\tan\theta_{0}}=\frac{\sqrt{2}ln2}{n\pi}\frac{R}{\Delta R}\frac{\lambda_{0}}{\sin\theta_{0}\tan\theta_{0}}$ (S6-2)

Eq. (S1-10) requires $\Delta R\ll R_{0}\cos^{2} \theta_{0}$. Assume an upper limit $\frac{\Delta R}{\left( R_{0}\cos^{2} \theta_{0} \right)}\leq1/3$, and $NA=n\sin\theta_{0}$, we have

$\Delta z=\frac{\sqrt{2}ln2}{n\pi}\frac{R}{\Delta R}\frac{\lambda_{0}}{\sin\theta_{0}\tan\theta_{0}}\geq\frac{3\sqrt{2}ln2n}{\pi}\frac{\lambda_{0}}{\cos\theta_{0}{NA}^{2}}\approx\frac{1.4\lambda_{0}}{{NA}^{2}}$ (S6-3)

Here, we have used $\cos\theta_{0}=0.89$ and $n=1.33$ in our experimental conditions. Eq. (S6-3) shows that the higher limit of the depth resolution of B^2^-SRS is about $\frac{1.4\lambda_{0}}{{NA}^{2}}$, which is close to the depth resolution of the C-SRS imaging ($\sim\frac{\sqrt{2}\lambda_{0}}{{NA}^{2}}$).

**S-7. Supplementary Movie S1 on 3D imaging of Brownian motions of 2** $\boldsymbol{\mu m}$ **PS beads in water using v-mode B^2^-SRS**

Movie S1 shows 3D Brownian motion of 2 $\mu m$ PS beads in water acquired with v-mode B^2^-SRS. The phantom volume measured is $46\times46\times7 {\mu m}^{3}$ ($128\times128\times5$ pixels). Through electronically varying the phase patterns on the SLM at a refresh rate of 60 Hz, we can monitor the 3D Brownian motions of PS beads in water with a volumetric frame rate of 10 vol/s. Figure S4 shows a snapshot of B^2^-SRS imaging obtained by rapid phase pattern projections on SLM. The 792 nm of the pump and 1041 nm of the Stokes beams are used to generate SRS signal of 2 $\mu m$ PS beads at Raman shift of 3050 cm^-1^. Time constant of a lock-in amplifier is set at 1 $\mu s$.


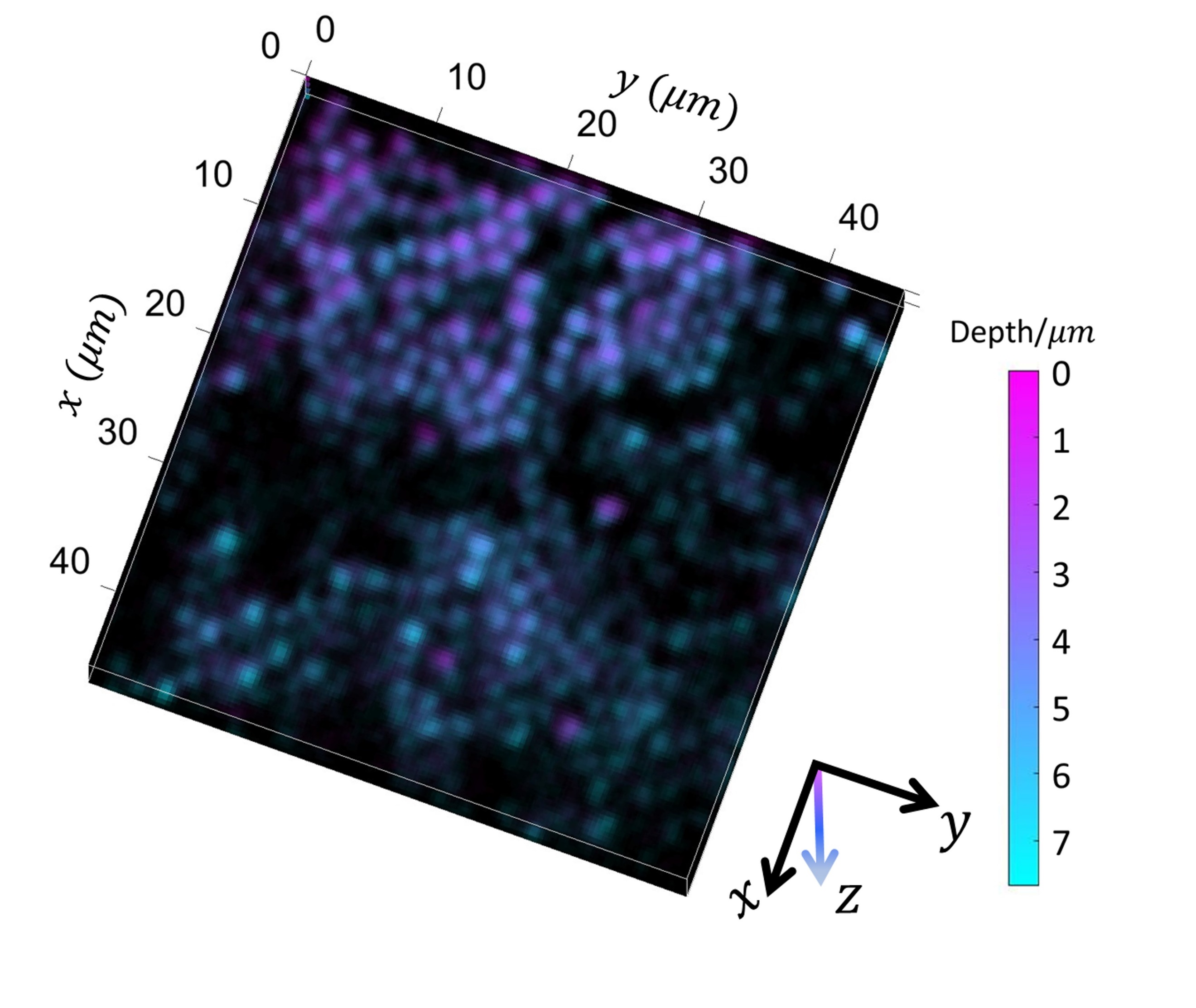


**Figure S4.** A snapshot of 3D Brownian motions of 2 $\mu m$ PS beads in water acquired by using the v-mode B^2^-SRS imaging.
